# Sexual dysfunction and its associated factors among reproductive-age women at Gurage Zone, Southern Ethiopia, 2023

**DOI:** 10.1186/s12889-023-16938-4

**Published:** 2023-10-18

**Authors:** Fentahun Tamene Zeleke, Semer Ezedin, Fentahun Aleminew, Kassa Genetu Alem, Daniel Tsega Tefera, Mebratu Demissie, Gudeta Beriso Jima, Fikremariam Endeshaw, Aynalem Belay, Alemitu Ayele, Demeke Andebet, Ambaye Minayehu Zegeye

**Affiliations:** 1 Department of Midwifery, College of Medicine and Health Sciences, Wolkite University, Wolkite, Ethiopia; 2 Wolkite University Specialized Hospital, Wolkite, Ethiopia; 3 Department of Midwifery, College of Medicine and Health Sciences, Bahir Dar University, Bahir Dar, Ethiopia; 4 Department of Midwifery, College of Medicine and Health Sciences, Dilla University, Dilla, Ethiopia; 5 College of Health Sciences, Department of Midwifery, Madawalabu University, Robe, Ethiopia; 6 Department of Midwifery, Hailu Alemu College, Gojjam, Ethiopia; 7 Department of Midwifery, College of Medicine and Health Sciences, Asossa University, Asossa, Ethiopia

**Keywords:** Female sexual dysfunction, Reproductive age women, Gurage zone, Ethiopia

## Abstract

**Introduction:**

Female sexual dysfunction is commonly neglected, under-investigated, and under-treated in Ethiopia. Therefore, this study aimed to determine the prevalence and its associated factors of female sexual dysfunction among reproductive-aged women at Gurage zone hospitals, in southern Ethiopia.

**Methods:**

An institutional-based cross-sectional study was conducted among 424 reproductive-age group women. A systematic random sampling method was employed and structured questionnaires were used to collect the data through a face-to-face interview. Data were entered into EpiData version 4.6 and analyzed by SPSS version 25.0. Descriptive statistics, and bivariable, and multivariable logistic regression were conducted. Statistical significance was declared at a *p*-value of < 0.05.

**Result:**

Four hundred two participants completed the interview with a response rate of 94.8%. Arousal dysfunction 91.0% and pain during sexual intercourse 39.3% were the most and the least prevalent domains of female sexual dysfunction respectively. Overall 32.1% of the respondents had female sexual dysfunction. Body mass index (AOR = 3.6; 95% CI: 1.2, 10.8), history of pelvic surgery (AOR = 3.5; 95% CI: 1.3, 9.2), marriage satisfaction (AOR = 3.9; 95% CI: 1.4, 1o.6), a satisfaction of spouses’ sex ability (AOR = 3.1; 95% CI: 1.2, 8.5), breastfeeding (AOR = 3.3; 95% CI: 1.6, 7.0), and mode of delivery [vaginal delivery with tear and episiotomy (AOR = 3.7; 95% CI: 1.6, 8.8), instrument assisted vaginal delivery (AOR = 7.0; 95% CI: 1.2, 39.8)], were statistically associated with female sexual dysfunction. All-encompassing professional counseling addressing psychological and interpersonal acts and weight management interventions are needed for couples to maintain sexual functioning.

## Plain language summary

Four hundred two participants completed the interview making the response rate 94.8%. About 247 (61.4%) respondents were under the age of 30 with a mean age of 28.14 ± 6.33 years. Arousal dysfunction was the most prevalent 366 (91.0%) domain of female sexual dysfunction while pain during sexual intercourse 158 (39.3%) was the least reported domain of female sexual dysfunction. Overall, in this study about 129 (32.1%) of the respondents had female sexual dysfunction. Body mass index, history of pelvic surgery, marriage satisfaction, satisfaction of spouses’ sex ability, breastfeeding7, and mode of delivery were statistically associated factors with female sexual dysfunction in multivariable analysis. All-encompassing professional counseling that addresses psychological and interpersonal acts and the importance of weight management interventions is needed for couples to maintain sexual functioning.

## Background

 Sexual life is a natural and complex element of human behavior, which is determined by numerous physiological, psychological, and social factors [1–3]. World Health Organization defines sexual health as “physical, emotional, mental, and social well-being concerning sexuality, not merely the absence of disease, dysfunction, or infirmity” [4]. It is thought that female sexual function is composed of a set of domains, including desire, genital excitation (lubrication), orgasm, satisfaction, and pain [5]. Thus, sexual dysfunction is a disturbance in these sex response cycles that do not allow the achievement of the expected outcome [6]. There are varieties of causal factors that lead to female sexual dysfunction (FSD). These include anatomical, hormonal, neurological, psychological, or sociocultural factors and medications or drug abuse [2, 7]. Besides these factors, sexual intercourse is not only influenced by the integrity of the genital tract but also by the limbic system and the excitation centers of the spine [8, 9]. An important element of sexual desire in women is reactive instead of spontaneous. As a result, women’s motivation and ability to find and respond to sexual excitement and subsequent sexual desire are crucial but complex [8–10]. In continuing relationships, a woman’s motivation appears to be largely influenced by her intimacy with her partner and her desire to improve it. It fits well with the way mental excitement finds the sexual stimulus and its context and poorly with changes in objective genital blood flow [9–13].

The prevalence of FSD varies by community, with the global prevalence ranging from 35.4 to 62.1% [6, 14, 15]. A systematic review and meta-analysis conducted in 2016 showed that the prevalence of FSD in 215,740 reproductive-age women over the world was 41% [16]. In the USA, 30 to 50% of women suffer from sexual dysfunction. Lack of interest in sex, inability to attain orgasm, pain during sex, and sex that is not pleasurable account for 27–32%, 22–28%, 8–21%, and 17–27% of all dysfunctions in the USA, respectively [17]. The prevalence of sexual dysfunction among reproductive-age women in Iran was found to be 52% [18]. According to a Nigerian cross-sectional survey, 89% of reproductive-age women have at least one form of sexual dysfunction [17].

Although sexual dysfunction is more common, only a small number of women seek counseling or therapy for it [7, 19, 20]. This is because of societal and cultural hurdles, taboos, and misconceptions about sexual concerns, particularly in Africa. Indeed, discussing sexual issues with children and adolescents is taboo in most African countries including Ethiopia [15, 18, 21].

Sexual dysfunction is typically multifaceted with socioeconomic, biological, medical, and psychological factors all playing a role [15, 17, 22, 23]. In addition, hormonal and physiological changes, cultural and ethical difficulties, religious views, misconceptions and fears, and the changing structure of women’s roles have all been documented to have an impact on women’s sexual lives [19].

Age, religion, and working hours of more than 8 per day were found to be significant related factors for FSD [20, 24–27]. Poor physical health, abortion, female genital mutilation, vaginal delivery with sutures, relationship unhappiness, and sexual assault were identified as risk factors for FSD in a study done in Germany [2]. Another study in China demonstrated that dissatisfaction with the spouse’s sexual ability, spouse’s sexual difficulties, dissatisfaction with married life, living in a rural area, chronic pelvic pain, chronic disease, previous pelvic surgery, vaginal delivery, and lower education were associated with FSD [22]. A longitudinal study of Iranian women found that the length of marriage, the presence of an episiotomy, and contraception methods were all factors associated with FSD [28]. A study conducted among Saudi Women identified that only age, low family income, and dissatisfaction with a spouse’s sexual ability sustained independent significant factors in a multivariate logistic regression analysis [25]. Studies conducted in Nigeria showed that age, years of relationship, number of children alive, and parity were significantly associated with FSD [6, 17]. However, other studies in Nigeria demonstrated that family income, being overweight or obese, being diabetic or hypertensive, religion, ethnic group, and educational qualification had no significant association with FSD [15, 17]. A study conducted in southwest Ethiopia among diabetic patients demonstrated that age, educational level, and physical activity were significantly associated with sexual dysfunction among diabetic patients [29]. Studies on FSD and its socio cultural behavior are lacking in Ethiopia, particularly at Gurage. Hence, the epidemiology of FSD and its associated factors in Ethiopian women is largely unknown. Understanding female sexual satisfaction and its associated factors would facilitate efforts to promote the quality of life among Ethiopian women.

Female sexual dysfunction has a detrimental impact on public health because it impairs the quality of life and physical well-being of women [17, 30, 31]. It hurts self-esteem and has put a strain on family relationships, causing frustration, agony, anxiety, and depression [17]. It also causes marital infidelity, which leads to an increase in the frequency of sexually transmitted illnesses, emotional suffering, and interpersonal relationships [17, 25, 32]. This is particularly true in African societies, where the majority of discussions concerning female sexual health are frowned upon [20, 21]. This is also obvious in learning institutions, where a lack of sex education, as well as religious and sociocultural factors, tend to influence sexual attitudes [24].

Women’s sexual dysfunction is frequently neglected, under-identified, and undertreated by healthcare professionals, despite its high prevalence and considerable impact on life [24, 26, 33]. Regardless of the numerous studies on FSD conducted around the world, this is not the case in Sub-Saharan Africa [34]. Existing studies, particularly in Ethiopia, are carried out in hospitals, primarily among people who have chronic illnesses [1, 29, 35]. It is a typical question about the extent of FSD and its associated factors among reproductive-aged women. Thus, this study aimed to determine the prevalence and its associated factors of FSD among reproductive-aged women attending Gurage zone hospitals, in southern Ethiopia.

## Methodology

### Study area and period

This study was conducted at governmental public hospitals in the Gurage zone, southern Ethiopia. Gurage Zone is one of the administrative zones of SNNPR in Ethiopia. It is divided into 16 districts and 5 town administrations where Wolkite town is the zone’s capital. Gurage Zone has a total population of 1,635,311 people, with 842,065 women and 793,246 men, according to the Ethiopian Central Statistical Agency’s 2017 population forecast [36]. The zone’s total population is served by five governmental public hospitals and two private hospitals. Five of the zone’s hospitals are primary care facilities, one is a general zonal hospital, and one is a university-specialized teaching hospital. The study was conducted from 09 to 2023 to 10 March 2023.

### Study design and population

An institution-based cross-sectional study was conducted at hospitals in the Gurage zone, southern Ethiopia. All reproductive-age women who visited hospitals in the Gurage zone during data collection time were the source population in this study.

### Inclusion and exclusion criteria

All married reproductive-aged women who visited Gurge zone hospitals during data collection were included in this study.

Women currently not living with the spouse, pregnant women, and women within 6 weeks of postpartum were excluded. Women who had undergone major pelvic surgery within the last 4 weeks or planned to undergo major pelvic surgery and women with a known mental disorder were also excluded from this study.

### Sample size determination

The sample size was determined by using a single population proportion formula with the assumption of a 95% confidence interval, 5% precision, and taking the prevalence of sexual dysfunction 50% due to the lack of previous local studies among this group of population

$$\mathrm n\;=\;\left[\left(\mathrm Z\frac{\mathrm a}2\right)^2\mathrm x\;\mathrm P\;\left(1-\mathrm p\right)\right]/d^2$$

$$n=\;\left[\left(1.96\right)^2x\;\left(0.5\right)\;\left(1-0.5\right)\right]/\left(0.05\right)^2$$

$$\mathrm n=\;\left[\left(1.96\right)^2\mathrm x\;\left(0.5\right)\;\left(0.5\right)\right]/\left(0.05\right)^2$$

$$\mathrm n\;=\;385$$

Where,

- ➢ ➝ n= sample size
- ➢ ➝ Z = critical value for normal distribution at 95% confidence level which equals 1.96 (z value at α = 0.05).
- *➢ ➝ P *= (Proportion of sexual dysfunction 50%).

- ❖ ➝ To compensate for non-response 10% was added making a total sample size of 424 reproductive-age women.

The sample size for the second objective was determined by Epi info software version 7.2 using significant factors from the study conducted in Cameroon and Saudi by considering the following assumptions: 95% confidence interval, 80% power, 1 ratio of unexposed to exposed (Table 1).

**Table 1:** Sample size calculation by taking significant factors from the previous study

Factor	Category	FSD		OR	Sample size with 10% non-response rate	Reference
		Yes	No			
Marital status	Married	3	18	3.5	132 + 14 = 146	[24, 25]
	Not married but in a relationship	82	140			
Chronic disease	Yes	28	21	1.4	296 + 30 = 326	
	No	143	213			
Satisfaction with spouse’s sexual ability	Dissatisfied	20	3	1.5	80 + 8 = 88	
	Fair/satisfied	100	77			

This is not an adequate sample as compared with the previous one. Thus the final sample size for this study was 424.

### Sampling technique and procedures

**Fig. 1:**
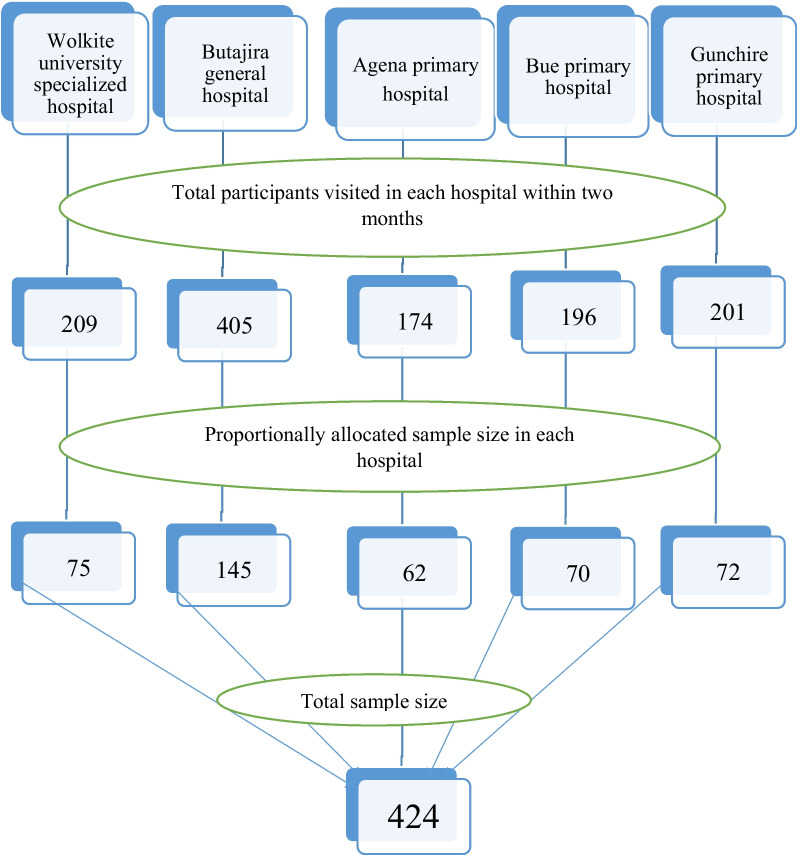
Proportional allocation and sampling procedure

Totally 1185 reproductive-aged women had visited family planning, gynecologic, and outpatient clinics of all governmental public hospitals in a Gurage zone within two months. Using this total population we distributed a calculated sample size with probability proportional allocation to the size of reproductive-age women in each hospital. Then after systematic random sampling method was used to select the study units in each hospital. With this method, we draw the sampling unit every 2 values, since the calculated k value was 2 which is obtained by dividing the total population by the total sample size (Fig. 1).

### Data collection method

A structured questionnaire was prepared according to the objectives of the study from previous relevant works of literature in the English language and adapted to the local context [6, 14, 15, 19, 21, 23–25]. Contextualizing the variables and translating the questionnaire into the local Amharic language were steps in the adaption process. The Amharic version was retranslated into English to ensure consistency. Additionally, face validity was checked. The questionnaire contains six parts including socio-demographic characteristics, gynecologic profile, obstetric profile, questions concerning general medical health and lifestyle, questions concerning the sexual relationship with spouse, and questions that were used to determine the female sexual function index. Twenty-three BSc holders collected data under the supervision of seven supervisors. Data were collected through a face-to-face interview in a separate room.

### Dependent variable

Prevalence of sexual dysfunction.

### Independent variable

The independent variables in this study were grouped into socio-demographic features, gynecologic factors, obstetric factors, general health conditions and lifestyle, and sexual life characteristics. Variables listed under socio-demographic features include age, religion, ethnicity, residence, educational level, occupation, housing situation, marriage duration, couples’ age differences, monthly family income, and BMI. The gynecologic-related factors include female genital mutilation, age at coitarche, genital tract infection, pelvic surgery, family planning method, menstrual cycle, and history of sexual assault. Variables grouped under obstetric factors include parity, number of live children, sexual resumption after delivery, breastfeeding, and mode of delivery. Variables included under general health conditions and lifestyle were chronic medical diseases, daily workload, and physical exercise. Lastly, variables under sexual life characteristics include marriage satisfaction, the spouse’s sexual difficulties, and satisfaction with the spouse’s sexual ability.

### Data measurement

The female sexual function index (FSFI) was used to assess sexual function, and it is a valid questionnaire with a high level of reliability ranging from 0.76 to 0.93 [15, 19, 37–40]. The FSFI as part of the questionnaire used in this study had 19 multiple-choice items and was designed to gather information on female sexual functioning during the previous four weeks. With this tool, the six main domains of sexual function including desire, arousal, lubrication, orgasm, satisfaction, and pain were assessed [37].

Questions 1, 2, 15, and 16 were assessed with a Likert scale from 1 to 5, while the others were scored on a six-point Likert scale ranging from 0 to 5. Then, each of the six domains of sexual function was calculated by summing each domain’s score and multiplying the result by the domain factors (Table 2).

**Table 2:** Score range and factor to calculate each domain’s score

Domain	Questions	Factor	Minimum score	Maximum score	Possible score range	References
Desire	1 and 2	0.6	1.2	6	1–5	[19, 42]
Arousal	3, 4, 5, and 6	0.3	0	6	0–5	
Lubrication	7, 8, 9, and 10	0.3	0	6	0–5	
Orgasm	11, 12, and 13	0.4	0	6	0–5	
Satisfaction	14, 15, and 16	0.4	0.8	6	0 or 1–5	
Pain	17, 18, and 19	0.4	0	6	0–5	

Participants were classed as having difficulties in that area if their domain scores were less than 4.28 on desire, 5.08 on arousal, 5.45 on lubrication, 5.05 on orgasm, 5.04 on satisfaction, and 5.51 on pain. The FSFI total score, which ranges from 2 to 36, is based on the sum of the six categories, and a cutoff score of 26 or less was used to identify women with sexual dysfunction [15, 19, 39, 41].

### Data quality control

The questionnaire was adapted from previous different kinds of literature. The pretest of the instrument was done before the actual data collection period at Durame General Hospital, which is out of the study area, among 5% [22] of the study participants. Accordingly, adjustments in the sequence and wording of the questionnaire were made based on the results of the pre-test. Two-day training about the objectives and process of the data collection was given to data collectors. Trained supervisors supervised the data collectors daily for the completeness and consistency of the filled questionnaires. In addition, the data was thoroughly cleaned and carefully entered into a computer for the beginning of the analysis.

### Data processing and analysis

Data were coded and entered into EpiData Manager version 4.6 and then exported to SPSS version 25.0 for analysis. During analysis, data was edited and cleaned for inconsistencies and analyzed using SPSS 25 statistical software. Descriptive statistics like frequencies and percentages were presented with texts, tables, and simple bar graphs. Bivariable logistic regression analysis was performed to see the association between each independent variable and the outcome variable. Independent variables with 𝑝-value of ≤ 0.25 in binary logistic regression and deemed important variables by the researcher were entered into multivariable logistic regressions for not to leave important variables. Multi-collinearity was checked by using the Variance Inflated Factor (VIF), which ranges from 1.010 to 1.199. Model fitness was checked using the Hosmer-Lemeshow test. Association was described using an adjusted odds ratio along with 95% CI and *p*-value < 0.05 was considered statistically significant.

## Result

### Socio-demographic characteristics

A total of 402 participants completed the interview with a response rate of 94.8%. The majority, 247 (61.4%) of the respondents were below age 30 with a mean age of 28.14 ± 6.33 years. About 232 (57.7%) and 146 (36.3%) of the participants were Islamic and Orthodox Tewahido Christianity religion followers respectively. Regarding their ethnic group, 293 (72.9%) of the respondents belonged to the Gurage ethnic group followed by Mareqo 40 (10%) ethnic groups. About 243 (60.4%) participants were urban residents, 145 (36.1%) completed their primary education, 205 (51%) participants had shared one bed with their babies, and about 291 (72.4%) were housewives. The mean age difference between couples was 7.8 ± 3.9 years. The length of marriage for the majority was below 5 years with the mean length of marriage 8.98 ± 7.49 years. The daily workload for 152 (37.8%) of the respondents was more than 8 h while only 23 (5.7%) had practiced regular physical exercise. The average monthly family income was 4864.9 ± 2510.3 Ethiopian birrs and the majority’s 261 (64.9%) monthly family income was below 5250 Ethiopian birrs (Table 3).

**Table 3:** Socio-demographic features of reproductive-age women in Gurage zone, southern Ethiopia from 09 January 2023 to 10 March 2023 (*N* = 402)

Characteristics	Category	Frequency	Percentage
Age category	< 30	247	61.4
	30–35	106	26.4
	36–40	31	7.7
	> 40	18	4.5
Religion	Muslim	232	57.7
	Orthodox Tewahido Christianity	146	36.3
	Other	24	6.0
Ethnicity	Gurage	293	72.9
	Mareqo	40	10.0
	Others	69	17.2
Occupation	Housewife	291	72.4
	Government employee	47	11.7
	Employed in the private sector	22	5.5
	Merchant	32	8.0
	Others	10	2.5
Educational status	Can’t read and write	105	26.1
	Primary	145	36.1
	Secondary	96	23.9
	College/University	56	13.9
Housing situation	Large house (more than one bedroom)	205	51.0
	Small house (One bedroom for them and their children)	197	49.0
Couples’ age difference	< 5	73	18.2
	5–10	252	62.7
	> 10	77	19.2
Length of marriage	< 5	138	34.3
	5–10	131	32.6
	> 10	133	33.1
Daily workload	Less than 8 h	250	62.2
	Above 8 h	152	37.8
Monthly family income	< 5250 EB	261	64.9
	≥ 5250 EB	141	35.1

### Gynecologic, obstetric, and general health profiles

The majority of 310 (77.1%) of the respondents had a history of female genital mutilation. The mean age of the respondents at Coitarche was 19.3 ± 3.1 years and 11(2.7%) of the respondents had a history of sexual assault. About 30 (7.5%) and 41 (10.2%) of the respondents had a history of genital tract infection and pelvic surgery within the previous 24 months respectively. One hundred thirty-two (32.8%) had utilized modern family planning methods within the last 12 months. The great majority 348 (86.6%) of the respondents had given birth before and 48 (11.9%) of the respondents had more than five children. Of those who delivered 199 (57.2%) delivered their last baby vaginally without tear/episiotomy and 247 (71%) were feed breast for their baby during data collection time. Twenty-eight (7.0%) of the participants had a history of chronic medical diseases within the previous 12 months and of these hypertension accounts for 15 (53.6%) followed by DM 11 (39.3%) and TB 2 (7.1%). Regarding participants’ body mass index about 73 (18.2%) and 77 (19.2%) of the respondents were underweighted and overweighed respectively (Table 4).

**Table 4:** Gynecologic, obstetric, and general health profiles of reproductive-age women in Gurage zone, southern Ethiopia from 09 January 2023 to 10 March 2023 (*N* = 402)

Characteristics	Category	Frequency	Percentage
Age at coitarche in years	< 18	130	32.3
	≥ 18	272	67.7
Feature of the menstrual cycle	Regular	345	85.8
	Irregular	57	14.2
Mode of delivery for the last baby	Vaginal delivery without tears/episiotomy	199	49.5
	Vaginal delivery with tears/episiotomy	88	21.9
	Instrumental delivery	18	4.5
	Cesarean section	43	10.7
	No delivery at all	54	13.4
Number of children	1–2	172	42.8
	3–5	128	31.8
	> 5	48	11.9
Type of utilized family planning method	COC	17	12.9
	Depo	58	43.9
	Implant/jadelle	40	30.3
	IUCD	13	9.8
	Others	4	3.0
Body mass index	18.5–24.9	252	62.7
	< 18.5	73	18.2
	25>=	77	19.2

### Marital and sexual life

**Fig. 2:**
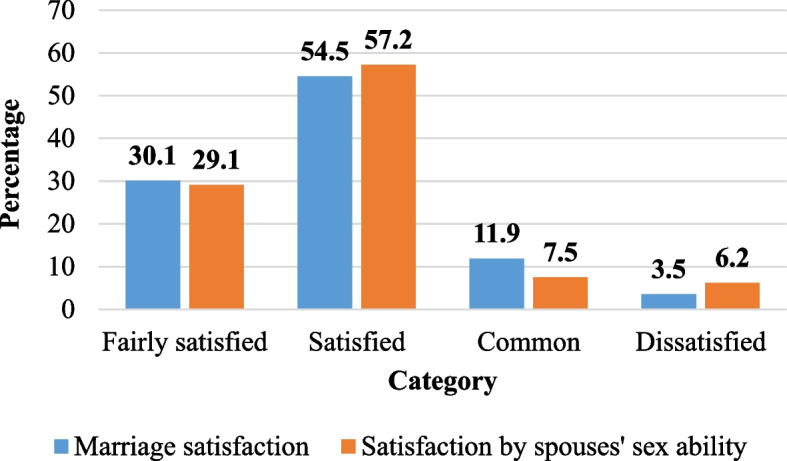
Marital and sexual status of reproductive-age women in Gurage zone, southern Ethiopia from 09 January 2023 to 10 March 2023 (*N* = 402)

About 62 (15.4%) of the respondents were dissatisfied with their marriage. Of those who delivered 305 (87.6%) participants resumed their sexual practice within 5–8 weeks. About 30 (7.5%) of the respondents were reported that their partners had diagnosed with sexual dysfunction and 55 (13.7%) were dissatisfied with their spouses’ sexual ability (Fig. 2).

### Female sexual dysfunction

**Fig. 3:**
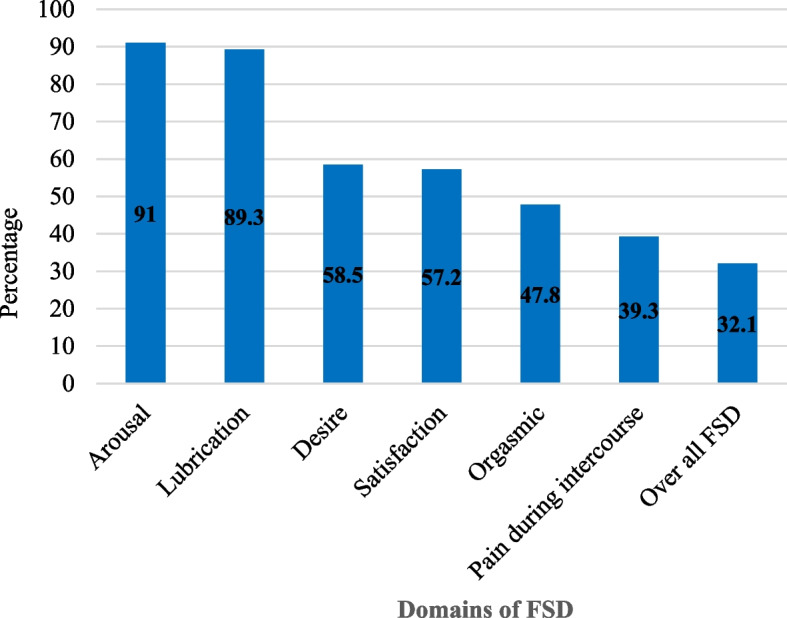
Prevalence of sexual dysfunction among reproductive-age women in Gurage zone, southern Ethiopia from 09 January 2023 to 10 March 2023 (*N* = 402)

In this study, arousal dysfunction was the most prevalent 366 (91.0%) domain of FSD while pain during sexual intercourse 158 (39.3%) was the least reported type of FSD. Overall, in this study about 129 (32.1%) of the respondents had FSD (Fig. 3).

### Factors associated with female sexual dysfunction

On bivariable logistic regression analysis body mass index, length of the marriage, couple’s age difference, history of genital tract infection, history of pelvic surgery, the feature of the menstrual cycle, daily workload, marriage satisfaction, a satisfaction of spouses’ sex ability, breastfeeding, housing situation, and mode of delivery were significantly associated with FSD (*p* < 0.05). Body mass index, length of the marriage, history of pelvic surgery, marriage satisfaction, the satisfaction of spouses’ sex ability, breastfeeding, and mode of delivery were continued statistically associated with FSD (*P* < 0.05) in multivariable analysis.

This study showed that the odds of FSD were 3.6 times (AOR = 3.62; 95% CI = 1.22, 10.77) higher among those overweighed respondents as compared to those who had normal body mass index. This is due to that increased BMI can result in lower body image, which can affect sexual functioning in significant ways. However, some studies argued this finding by concluding that there is no difference in mean FSFI scores between subjects with normal BMI and overweight ones [42].

Participants who had a history of pelvic surgery were 3.5 times (AOR = 3.46; 95% CI = 1.3, 9.2) more likely to develop FSD as compared to their counterparts. This can due to surgery through the vagina can leave scar tissue that could modify the sexual function of patients. On the other hand, dissection near the hypogastric plexus in the abdominal approach can also have an impact on sexual function [43].

Participants who had chronic medical diseases were 10.6 times (AOR = 10.58; 95% CI = 2.58, 43.34) more likely to develop FSD as compared to their counterparts. Chronic medical diseases can affect sexual life directly by disrupting normal functioning including body hormones and indirectly by bringing a reduction of self-image, depression, and body impairment. Those participants who were dissatisfied with their marriage were 3.9 times (AOR = 3.88; 95% CI = 1.42, 10.60) more likely to develop FSD as compared to those who were satisfied with their marriage. Those participants who were dissatisfied with their spouses’ sexual ability were 3.1 times (AOR = 3.15; 95% CI = 1.66, 8.50) more likely to develop FSD as compared to those who were satisfied with their spouses’ sexual ability. Those participants who fed breasts to their children were 3.3 times (AOR = 3.32; 95% CI = 1.58, 6.98) more likely to develop FSD as compared to those who did not breastfeed. Those participants who delivered their last child with tears/episiotomy and instrument were 3.7 (AOR = 3.72; 95% CI = 1.57, 8.80) and 7.0 (AOR = 7.04; 95% CI = 1.24, 39.8) times more likely to develop FSD as compared to those who delivered vaginally without tears/episiotomy respectively (Table 5).

**Table 5:** Factors associated with FSD among reproductive-age women in Gurage zone, southern Ethiopia from 09 January 2023 to 10 March 2023 (*N* = 402)

Variables	Category	FSD		Bivariable Analysis		Multivariable Analysis	
		Yes	No	OR	95% CI	AOR	95% CI
Body mass index	18.5–24.9	58	194	1	1	1	1
	25>=	47	30	5.2	3.0–9.0 **	3.6	1.2–10.8 *
Couples’ age difference	< 5	14	59	1	1	1	1
	5–10	82	170	2.0	1.0-3.9 *	0.91	0.4–2.3
	> 10	33	44	3.2	1.5–6.6 **	1.3	0.4–4.3
History of STI	Yes	17	13	3.0	1.4–6.4 **	0.82	0.3–2.6
	No	112	260	1	1	1	1
History of pelvic surgery	Yes	26	15	4.3	2.2–8.5 **	3.5	1.3–9.2 *
	No	103	258	1	1	1	1
Menstrual cycle	Regular	103	242	1	1	1	1
	Irregular	26	31	2.0	1.1–3.5*	1.7	0.7-4.0
Daily workload	< 8 h	67	183	1	1	1	1
	> 8 h	62	90	1.9	1.2–2.9**	1.3	0.6–2.5
Marriage satisfaction	Satisfied	84	256	1	1	1	1
	Dissatisfied	45	17	8.0	4.4–14.8 **	3.9	1.4–10.6 **
Satisfaction with spouses’ sexual ability	Satisfied	94	253	1	1	1	1
	Dissatisfied	35	20	4.7	2.6–8.6 **	3.1	1.2–8.5 *
Breastfeeding	Yes	96	151	2.4	1.5–3.7 **	3.3	1.6-7.0 **
	No	33	122	1	1	1	1
Housing situation	Large house	43	162	2.9	1	1	1
	Small House	86	111	1	1.9–4.5 **	1.4	0.6–3.1
Mode of delivery	VDWOT&E	54	145		1	1	1
	VDWT&E	40	48	2.2	1.2–3.8 **	3.7	1.6–8.8 **
	Instrumental	14	4	9.4	3.0-29.8 **	7.0	1.2–39.8 *

* Significant (*p* < 0.05); ** highly significant (*p* < 0.01); VDWOT&E-vaginal delivery without tear and episiotomy; VDWT&E- vaginal delivery with tear and episiotomy

## Discussion

### Prevalence of female sexual dysfunction

This study shows that arousal dysfunction 91.0% (87.8-93.6%) was the most prevalent domain of FSD. A cross-sectional study conducted in Nigerian and Iranian supports this finding [18, 23, 44]. This result is inconsistent with a finding found from a similar study conducted among Iranian women in which sexual desire was the most frequent domain of FSD [14]. Overall, in this study 32.1% (27.5-36.9%) of the respondents had FSD. This finding is lower than a study conducted on Iranian and Turkish women that shows 85.95% and 74.3% of participants had FSD respectively [19, 44]. This difference could be because of differences in the study population characteristics. Participants in the current study were all reproductive-aged women whereas the previous studies had conducted only among post-partum women. Evidence showed that FSD is more prevalent among this group of population (women in the post-partum period) as compared to the general reproductive-aged group [19]. It is also lower than a study conducted in Egypt that shows 50.1% of the respondents had FSD [20]. This difference could be due to differences in determining cutoff points for FSD. Our study declared FSD when the FSFI score was less than 26 whereas the previous study considered women with FSFI scores less than 23 had FSD. Our study finding was also lower than the study conducted on Singaporean and Iranian women which shows 43.2% and 62.1% of women had faced FSD [45]. This could be due to differences between participants’ characteristics. In previous studies, women beyond reproductive age were included which might increase the FSD prevalence [14, 45].

### Factors associated with female sexual dysfunction

This study showed that the odds of FSD were 3.6 times (AOR = 3.62; 95% CI = 1.22, 10.77) higher among those overweighed respondents as compared to those who had normal body mass index. Similarly, several studies reported the existence of an association between obesity and sexual inactivity. A case-control study conducted in Iran observed particularly that there was a strong and inverse correlation between BMI and arousal, lubrication, and orgasm [46–48]. Inconsistent with our study finding some studies had failed to show the association between FSD and BMI [49, 50]. Another factor influencing female sexual function was the length of marriage. In the present study participants who had a history of pelvic surgery were 3.5 times (AOR = 3.46; 95% CI = 1.3, 9.2) more likely to develop FSD as compared to their counterparts. This finding is supported by a study conducted in Beijing, China [22]. The possible explanation could be due to damage to the pelvic nerves or blood vessels during the procedure, inflammation, and fibrosis after the surgery, and mental issues related to the surgery. Supporting the result of a study conducted in China [22] our study finding revealed that participants who were dissatisfied with their marriage were 3.9 times (AOR = 3.88; 95% CI = 1.42, 10.60) more likely to develop FSD as compared to those who were satisfied with their marriage. This is due to that; sexual intercourse/satisfaction is a combined effect of physical, psychological, and social readiness. According to the present study participants who were dissatisfied with their spouses’ sexual ability were 3.1 times (AOR = 3.15; 95% CI = 1.66, 8.50) more likely to develop FSD as compared to those who were satisfied with their spouses’ sexual ability. Other pieces of evidence conducted in different areas also confirmed this finding [51, 52]. As the spouse’s sexual ability decreases female affection toward the spouse might be affected which in turn decreases female sexual libido even exceeding the influence of hormones [22]. The current study showed that women who fed breasts to their children were 3.3 times (AOR = 3.32; 95% CI = 1.58, 6.98) more likely to develop FSD as compared to those who were satisfied by their spouses’ sexual ability. Studies conducted in Brazil, and Hungary confirmed the significant association between breast-feeding and FSD [53, 54]. Another study also showed that sexual activity in lactating women resumes later than those who do not breastfeed and they complained of more pain during intercourse than other non-lactating women [55–57]. In contrast to this, a study conducted in Iran showed the presence of a direct correlation between breastfeeding and increased desire [58]. This could be due to an increment of prolactin and decreased level of estrogen and progesterone hormones that lead to vaginal dryness, atrophy, and reduction in the level of vaginal lubrication causing dyspareunia [57, 59]. The result of this study showed that participants who delivered their last child with tears/episiotomy and instrument were 3.7 (AOR = 3.72; 95% CI = 1.57, 8.80) and 7.0 (AOR = 7.04; 95% CI = 1.24, 39.8) times more likely to develop FSD as compared to those who delivered vaginally without tears/episiotomy respectively. Similarly, a study conducted on Spanish women showed the existence of a significant association between instrumental delivery and FSD [60]. Similarly, studies demonstrated that the association between instrumental delivery and FSD due to the increased risk of injuries that may occur with this type of intervention during delivery [61, 62]. Another study also reported that women with medio-lateral episiotomy were experienced decreased desire, arousal, and vaginal lubrication probably due to fears of perineal damage, which may lead to dissatisfaction as well as anorgasmia [63]. On the other hand, some studies show the absence of a direct association between episiotomy and FSD rather it protects by reducing the risk of third and 4th degree tears, which are causes of sexual dysfunction [26, 64, 65].

## Conclusion

Four hundred-two participants completed the interview with a response rate of 94.8%. The majority, 247 (61.4%) of the respondents were below age 30 with a mean age of 28.14 ± 6.33 years. Arousal dysfunction was the most prevalent 366 (91.0%) domain of FSD while pain during sexual intercourse 158 (39.3%) was the least reported type of FSD. Overall, about 129 (32.1%) of the respondents had FSD. Body mass index, history of pelvic surgery, marriage satisfaction, satisfaction of spouses’ sex ability, breastfeeding, and mode of delivery were factors statistically associated with FSD in multivariable analysis.

### Recommendations

Therefore, all-encompassing professional counseling is needed for couples about postpartum sexuality and the importance of weight management interventions as a strategy for maintaining sexual functioning. Additionally, professional support and education about psychological and interpersonal acts are needed for those who face a problem with their marriage and spouses’ sexual ability. These can be attained by creating a smooth and private environment for such in clinical practice. It is also recommended that responsible professionals and government bodies develop uniform guidelines to decrease technical/practical errors that might lead to sexual dysfunction after pelvic surgery.

### Strengths of the study

Being one of a few studies that address neglected and under-investigated topics in Ethiopia is the strength of this study.

### Limitations of the study

Although their confidentiality was reassured since sexuality is a sensitive issue, respondents may feel that their privacy was violated. As a result, the tendency to hold back or give false information could be a limitation of this study.

## Data Availability

The data sets used in this study were available from the corresponding author when reasonably requested.

## References

[1] Fanta T, Haile K, Abebaw D, Assefa D, Hibdye G (2018). Assessment of sexual dysfunction and associated factors among patients with schizophrenia in Ethiopia, 2017. BMC Psychiatry.

[2] McCool-Myers M, Theurich M, Zuelke A, Knuettel H, Apfelbacher C (2018). Predictors of female sexual dysfunction: a systematic review and qualitative analysis through gender inequality paradigms. BMC Womens Health.

[3] Camara A, Tounkara TM, Delamou A, Baldé R, Leno NN, Kuotu GC (2021). Prevalence and risk factors of female sexual dysfunction among women infected with HIV in Conakry. Clin Epidemiol Global Health.

[4] Organization WH (2006). Defining sexual health: report of a technical consultation on sexual health, 28–31 January 2002.

[5] Burri A, Hilpert P, Spector T (2015). Longitudinal evaluation of sexual function in a cohort of pre- and Postmenopausal women. J Sex Med.

[6] Adebusoye L, Ogunbode O, Owonokoko M, Ogunbode A, Aimakhu C (2020). Factors associated with sexual dysfunction among female patients in a Nigerian ambulatory primary care setting. Annals of Ibadan Postgraduate Medicine.

[7] Filocamo MT, Serati M, Li Marzi V, Costantini E, Milanesi M, Pietropaolo A (2014). The female sexual function index (FSFI): linguistic validation of the Italian version. J Sex Med.

[8] Berman JR, Berman LA, Werbin TJ, Goldstein I (1999). Female sexual dysfunction: anatomy, physiology, evaluation and treatment options. Curr Opin Urol.

[9] Fajewonyomi BA, Orji EO, Adeyemo AO (2007). Sexual dysfunction among female patients of reproductive age in a hospital setting in Nigeria. J Health Popul Nutr.

[10] Goldstein I (2000). Female sexual arousal disorder: new insights. Int J Impot Res.

[11] Berman JR, Goldstein I (2001). Female sexual dysfunction. Urologic Clin.

[12] Laumann EO, Paik A, Rosen RC (1999). Sexual dysfunction in the United States: prevalence and predictors. JAMA.

[13] Berman JR, Adhikari SP, Goldstein I (2000). Anatomy and physiology of female sexual function and dysfunction. Eur Urol.

[14] Jafarzadeh Esfehani R, Fazel N, Dashti S, Moshkani S, Haghighi Hasanabad F, Foji S (2016). Female sexual dysfunction and its associated risk factors: an epidemiological study in the North-East of Iran. J Midwifery Reproductive Health.

[15] Nwagha U, Oguanuo T, Ekwuazi K, Olubobokun T, Nwagha T, Onyebuchi A (2014). Prevalence of sexual dysfunction among females in a university community in Enugu, Nigeria. Niger J Clin Pract.

[16] McCool ME, Zuelke A, Theurich MA, Knuettel H, Ricci C, Apfelbacher C (2016). Prevalence of female sexual dysfunction among premenopausal women: a systematic review and meta-analysis of observational studies. Sex Med Reviews.

[17] Olugbenga-Bello A, Adebayo K, Goodman O, Oke O, Olukunle T (2020). Prevalence and socio-demographic determinants of sexual dysfunction among Married Women of Reproductive Age Group in South West Nige-Ria. J Reprod Med Gynecol Obstet.

[18] Ghiasi A, Keramat A (2018). Prevalence of sexual dysfunction among reproductive-age women in Iran: a systematic review and meta-analysis. J Midwifery Reproductive Health.

[19] Khajehei M, Doherty M, Tilley PJM, Sauer K (2015). Prevalence and risk factors of sexual dysfunction in Postpartum Australian Women. J Sex Med.

[20] El-Kashif M, El-tahry SE (2019). A study of sexual dysfunction and its associated factors among women of childbearing age, Egypt. J Nurs Educ Pract.

[21] Afefy N (2015). Factors associated with female sexual problems among women attending Cairo University Hospital. J Biol Agriculture Healthcare.

[22] Lou W-J, Chen B, Zhu L, Han S-M, Xu T, Lang J-H (2017). Prevalence and factors associated with female sexual dysfunction in Beijing, China. Chin Med J.

[23] Ogunbode O, Aimakhu C, Ogunbode A, Adebusoye L, Owonikoko K (2019). Sexual dysfunction among women in a Nigerian gynecological outpatients unit. Trop J Obstet Gynecol.

[24] Halle-Ekane GE, Timti LF, Tanue EA, Ekukole CM, Yenshu EV (2021). Prevalence and Associated factors of female sexual dysfunction among sexually active students of the University of Buea. Sex Med.

[25] Madbouly K, Al-Anazi M, Al-Anazi H, Aljarbou A, Almannie R, Habous M (2021). Prevalence and predictive factors of female sexual dysfunction in a sample of Saudi Women. Sex Med.

[26] Gutzeit O, Levy G, Lowenstein L (2020). Postpartum female sexual function: risk factors for postpartum sexual dysfunction. Sex Med.

[27] Holanda JBL, Abuchaim ESV, Coca KP, Abrão ACFV (2014). Sexual dysfunction and associated factors reported in the postpartum period. Acta Paulista De Enfermagem.

[28] Banaei M, Moridi A, Dashti S (2018). Sexual dysfunction and its Associated factors after delivery: longitudinal study in Iranian women. Mater Sociomed.

[29] Asefa A, Nigussie T, Henok A, Mamo Y (2019). Prevalence of sexual dysfunction and related factors among Diabetes Mellitus patients in Southwest Ethiopia. BMC Endocr Disorders.

[30] Zhang H, Fan S, Yip PS (2015). Sexual dysfunction among reproductive-aged Chinese married women in Hong Kong: prevalence, risk factors, and associated consequences. J Sex Med.

[31] Wolpe RE, Zomkowski K, Silva FP, Queiroz APA, Sperandio FF (2017). Prevalence of female sexual dysfunction in Brazil: a systematic review. Eur J Obstet Gynecol Reproductive Biology.

[32] Carreiro AV, Micelli LP, Sousa MH, Bahamondes L, Fernandes A (2016). Sexual dysfunction risk and quality of life among women with a history of Sexual Abuse. Int J Gynecol Obstet.

[33] Mateu Arrom L, Girabent-Farrés M, González M, Palou J, Errando-Smet C, Ramírez-García I (2021). Development and validation of a short version of the female sexual function index in the Spanish population. BMC Womens Health.

[34] Teuwafeu D, Ashuntantang G, Essi M, Kaze F, Maimouna M, Balepna J (2016). Sexual function and correlates in women undergoing maintenance hemodialysis in Cameroon: a multi-centric study. Open Urol Nephrol J..

[35] Ejigu AK, Zewlde KH, Muluneh NY, Seraj ZR, GebreLibanos MW, Bezabih YH (2019). Sexual dysfunction and associated factors among patients with Epilepsy at Amanuel Mental Specialty Hospital, Addis Ababa–Ethiopia. BMC Neurol.

[36] Shitu S, Abebe H, Adane D, Wassie A, Mose A, Yeshaneh A (2021). Knowledge of neonatal danger signs and associated factors among husbands of mothers who gave birth in the last 6 months in Gurage Zone, Southern Ethiopia, 2020: a community-based cross-sectional study. BMJ Open.

[37] Burri A, Cherkas L, Spector T (2010). Replication of psychometric properties of the FSFI and validation of a modified version (FSFI-LL) assessing lifelong sexual function in an unselected sample of females. J Sex Med.

[38] Rosen CB, Heiman J, Leiblum S, Meston C, Shabsigh R, Ferguson D, D’Agostino R (2000). The female sexual function index (FSFI): a multidimensional self-report instrument for the assessment of female sexual function. J Sex Marital Ther.

[39] Meston CM (2003). Validation of the female sexual function index (FSFI) in women with female orgasmic disorder and women with hypoactive sexual desire disorder. J Sex &Marital Therapy.

[40] Maroufizadeh S, Riazi H, Lotfollahi H, Omani-Samani R, Amini P (2020). The 6-item female sexual function index (FSFI-6): factor structure, reliability, and demographic correlates among infertile women in Iran. Middle East Fertility Society Journal.

[41] Wiegel M, Meston C, Rosen R (2005). The female sexual function index (FSFI): cross-validation and development of clinical cutoff scores. J Sex Marital Ther.

[42] Javadnoori M, Faridi H, Najar S (2015). The relationship between obesity/overweight and female sexual function. Maturitas.

[43] Shiozawa T, Huebner M, Hirt B, Wallwiener D, Reisenauer C (2010). Nerve-preserving sacrocolpopexy: anatomical study and surgical approach. Eur J Obstet Gynecol Reproductive Biology.

[44] Alidost F, Pakzad R, Dolatian M, Abdi F (2021). Sexual dysfunction among women of reproductive age: a systematic review and meta-analysis. Int J Reproductive Biomed.

[45] Logan S, Thu WPP, Ho K, Cauley JA, Kramer MS, Yong E-L (2021). Sexual inactivity and sexual dysfunction in midlife Singaporean women: a prospective cross-sectional study of prevalence and risk factors. Maturitas.

[46] Faubion SS, Fairbanks F, Kuhle CL, Sood R, Kling JM, Vencill JA (2020). Association between body mass index and female sexual dysfunction: a cross-sectional study from the data registry on experiences of aging, menopause, and sexuality. J Sex Med.

[47] Mozafari M, Khajavikhan J, Jaafarpour M, Khani A, Direkvand-Moghadam A, Najafi F (2015). Association of body weight and female sexual dysfunction: a case-control study. Iran Red Crescent Med J.

[48] Gonenir-Erbay L, Ozlu M, Sahin I, Evren B, Kayaalp C, Karlidag R. The effect of body mass index on the sexual functions of morbidly obese female patients. Dusunen Adam J Psychiatry Neurol Sci. 2017;30:338–43. 10.5350/DAJPN2017300408.

[49] Faridi H, Najar S, Javadnoori M (2013). The relationship between body mass index and women’s sexual function. Iran J Obstet Gynecol Infertility.

[50] Erbil N (2013). The relationships between sexual function, body image, and body mass index among women. Sex Disabil.

[51] Fugl-Meyer A, Sjogren K (1999). Sexual disabilities, problems and satisfaction in 18-74-year-old swedes. Scand J Sexol.

[52] Jiann BP, Su CC, Yu CC, Wu TT, Huang JK (2009). Risk factors for individual domains of female sexual function. J Sex Med.

[53] Holanda JBL, Richter S, Campos RB, Trindade RFC, Monteiro JCS, Gomes-Sponholz FA. Relationship of thetype of breastfeeding in the sexual function of women. Rev Latino-Am Enfermagem. 2021;29:e3438. 10.1590/1518-8345.3160.3438. 10.1590/1518.8345.3160.3438 PMC8294779 34287538

[54] Szöllősi K, Szabó L (2021). The association between infant feeding methods and female sexual dysfunctions. Breastfeed Med.

[55] Connolly A, Thorp J, Pahel L (2005). Effects of pregnancy and Childbirth on postpartum sexual function: a longitudinal prospective study. Int Urogynecol J.

[56] De Judicibus MA, McCabe MP (2002). Psychological factors and the sexuality of pregnant and postpartum women. J Sex Res.

[57] Heidari M, Merghati Khoei E, Kiani A (2009). A study of the relationship between sexual activity and breastfeeding. J Mazandaran Univ Med Sci.

[58] Anbaran ZK, Baghdari N, Pourshirazi M, Karimi FZ, Rezvanifard M, Mazlom SR (2015). Postpartum sexual function in women and infant feeding methods. High Educ.

[59] Avery MD, Duckett L, Frantzich CR (2000). The experience of sexuality during breastfeeding among primiparous women. J Midwifery Women’s Health.

[60] Hidalgo-Lopezosa P, Pérez-Marín S, Jiménez-Ruz A, López-Carrasco JdlC, Cubero-Luna AM, García-Fernández R (2022). Factors associated with postpartum sexual dysfunction in Spanish women: a cross-sectional study. J Personalized Med.

[61] Dağli E, Kul Uçtu A, Özerdoğan N. Sexual dysfunction in the postpartum period: Its relationship with postpartum depression and certain other factors. Perspect Psychiatr Care. 2021;57:604–9. 10.1111/ppc.12583. 10.1111/ppc.12583 32677049

[62] García-Mejido JA, Idoia‐Valero I, Aguilar‐Gálvez IM, Borrero González C, Fernández‐Palacín A, Sainz JA (2020). Association between sexual dysfunction and avulsion of the levator ani muscle after instrumental vaginal delivery. Acta Obstet Gynecol Scand.

[63] Eid M, Sayed A, Abdel-Rehim R, Mostafa T (2015). Impact of the mode of delivery on female sexual function after Childbirth. Int J Impot Res.

[64] Banaei M, Moridi A, Dashti S (2018). Sexual dysfunction and its associated factors after delivery: a longitudinal study in Iranian women. Materia socio-medica.

[65] Simic M, Cnattingius S, Petersson G, Sandström A, Stephansson O (2017). Duration of second stage of labor and instrumental delivery as risk factors for severe perineal lacerations: population-based study. BMC Pregnancy Childbirth.

